# LucidDraw: Efficiently visualizing complex biochemical networks within MATLAB

**DOI:** 10.1186/1471-2105-11-31

**Published:** 2010-01-15

**Authors:** Sheng He, Juan Mei, Guiyang Shi, Zhengxiang Wang, Weijiang Li

**Affiliations:** 1 The Key Laboratory of Industrial Biotechnology, Ministry of Education, Jiangnan University, Wuxi 214122, China; 2 School of Biotechnology, Jiangnan University, Wuxi 214122, China; 3 School of Computer Science, Jiangsu Teachers University of Technology, Changzhou 213001, China

## Abstract

**Background:**

Biochemical networks play an essential role in systems biology. Rapidly growing network data and versatile research activities call for convenient visualization tools to aid intuitively perceiving abstract structures of networks and gaining insights into the functional implications of networks. There are various kinds of network visualization software, but they are usually not adequate for visual analysis of complex biological networks mainly because of the two reasons: 1) most existing drawing methods suitable for biochemical networks have high computation loads and can hardly achieve near real-time visualization; 2) available network visualization tools are designed for working in certain network modeling platforms, so they are not convenient for general analyses due to lack of broader range of readily accessible numerical utilities.

**Results:**

We present LucidDraw as a visual analysis tool, which features (a) speed: typical biological networks with several hundreds of nodes can be drawn in a few seconds through a new layout algorithm; (b) ease of use: working within MATLAB makes it convenient to manipulate and analyze the network data using a broad spectrum of sophisticated numerical functions; (c) flexibility: layout styles and incorporation of other available information about functional modules can be controlled by users with little effort, and the output drawings are interactively modifiable.

**Conclusions:**

Equipped with a new grid layout algorithm proposed here, LucidDraw serves as an auxiliary network analysis tool capable of visualizing complex biological networks in near real-time with controllable layout styles and drawing details. The framework of the algorithm enables easy incorporation of extra biological information, if available, to influence the output layouts with predefined node grouping features.

## Background

The prevalence of computer-aided technologies for modeling large-scale biochemical networks causes a strong demand on visualization tools for intuitive presentation of the complex network structures. The key part of drawing a network is to place nodes in low dimensional (mostly, 2D) space such that the geometric distances between nodes reflect topological proximities described by the network. For very large complex networks involving many thousands of nodes, drawings may aim at grasping the global features, or macro characteristics, of the whole networks [1,2], the network details are often not readable. In contrast, a typical biochemical network like a metabolic network has some hundreds of nodes, which needs the visualization to clearly show both the global features (modules) and all individual links. To meet the needs, grid layout methods are developed recently and shown to have advantages in generating compact layouts with biologically comprehensible modules of biochemical networks [3-8].

A main issue of grid layout methods is the high computational cost, which seriously limits the applications. Miyano and co-workers proposed a method termed sweep calculation to speed up layout process [6]. Biological attributes of nodes as extra input are also used to reduce the search space and yield biologically interesting layouts [3-5]. Barsky *et al*. use similar strategy in their software Cerebral in which nodes are placed in predefined layers according to the subcellular localizations. They also use a technique to bundle edges connected to hub nodes and improve visual effect dramatically when high degree nodes are present [3]. Recently, Cerebral is developed further as a new visualization tool for analyzing experimental data in the context of an interaction graph model [9].

Extra biological attributes like subcellular localizations can be employed as constraints of node positions and consequently decrease the computational complexity substantially. In certain cases this helps generate high quality layouts [3-5]. Nonetheless, the use of such information is confined by several factors: 1) the extra information is often unavailable or incomplete; 2) it is rather artificial to decide how to arrange the layout areas allocated for nodes with different attributes; 3) when the number of nodes with some attribute is large, good placement of these nodes relies merely on the topology. To this end, speeding up node placement without additional constraints remains still an essential problem, which is the first motivation of this work.

As more and more interests are attracted on deep research of network properties, there arises another demand for automatic visualization as an auxiliary analysis tool. Available drawing tools for biochemical networks are designed to work in certain network modeling platforms such as Cytoscape [10], PATIKA [11], VisANT [12], Cell Illustrator [13,14], and CADLIVE [15,16]. Because these modeling platforms are designed for specific purposes, most network analysis related numerical utilities are not provided. In this respect, a drawing tool accessible within a more versatile numerical software environment will be convenient. For example, integrated in the Bioinformatics Toolbox of MATLAB, GraphViz http://www.graphviz.org provides researchers a way to visualize networks while making use of powerful numerical analysis functions of MATLAB. However, the implemented general graph drawing algorithms of GraphViz are usually not adequate to produce satisfactory drawings for complex biochemical networks. This is another motivation of this work.

In this paper, we present our solution, LucidDraw, for easy and quick visualization of complex biochemical networks. The tool is powered by a new grid layout algorithm and accessible from within MATLAB.

## Implementation

### The cost function and the weight matrix

A network layout is a configuration of the nodes and edges properly placed on a 2D plane. Generally, all nodes are represented as points without regard to their sizes and all edges are drawn as straight lines. Under such a drawing convention, a layout is fully described by the nodes' coordinates, denoted by **R **= (**r**_1_, **r**_2_, ..., **r**_*n*_), where *n *is the number of nodes and **r**_*i *_= (*x*_*i*_, *y*_*i*_) the coordinates. Because nodes are placed on grid points, all *x*_*i *_and *y*_*i *_are forced to be integers.

To determine the coordinates, we use a widely adopted method that treats nodes as interacting particles, and the layout quality is evaluated by a cost function that is defined as the total interaction energy of all pairs of the nodes with lower costs corresponding to better layouts. Following Li and Kurata [7], we use the cost function given by(1)

where *w*_*ij *_is the interaction weight of nodes *i *and *j*, which describes the way nodes interplay. The weights between all node pairs constitute the weight matrix. The term *d*_*ij *_is the Manhattan distance between nodes *i *and *j*. For detailed explanations about the design principles of the cost function, please refer to Ref [7].

**Figure 1:**
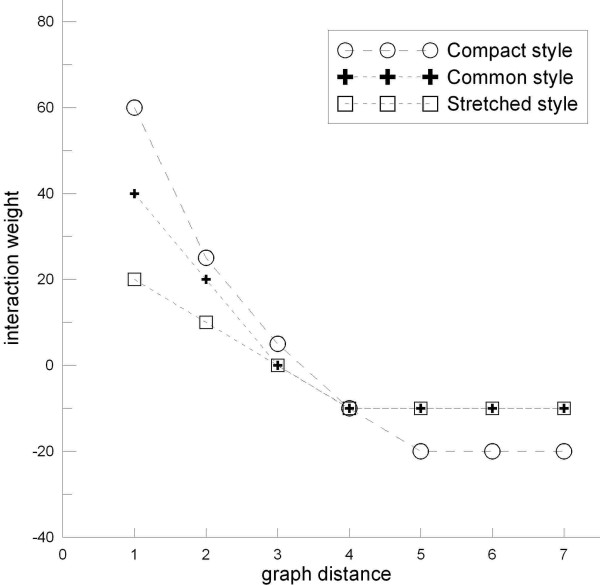
**Determination of interaction weights according to graph distances**. An evaluation scheme of the weight matrix corresponds to a style of layout. Three styles are shown here: 1) the common style, where weights are set to roughly balance the attractive and repulsive interactions, consequently nodes are placed relatively even in the layout area while modules are separated clearly; 2) the compact style, where attractive interactions are dominant, so nodes are located closely in a tight space, some modules may be not well separated; 3) the stretched style, where attractions are relatively weak, which makes modules distribute far from each other.

There are unlimited possibilities to choose detailed weight matrices. A convenient way is to evaluate the weight matrix according to the graph distances (i.e., shortest paths). Denote *L*_*ij *_the graph distance between nodes *i *and *j*, we set *w*_*ij *_= χ (*L*_*ij*_), where χ is some integer functions. By extensive experiments, we found three χ functions are suitable for typical biochemical networks. The corresponding layout styles are called common, compact, and stretched styles (Figure 1). The layout algorithm itself does not confine the weight matrix. Even when a predefined weight matrix is chosen, there is still room for users to modify some weights as wish. This provides flexible ways to use the method. For example, if two nodes are known *a priori *to belong to the same module and therefore hoped to be placed closely, one may add an extra positive value to the corresponding weight. See the Results section for an example.

### The layout algorithm

The layout algorithm aims to find the best layout by optimizing the cost function, which can be described as follows:

   Set **R **to a random layout

   Repeat the following steps for *niter *times

      Generate **R' **by perturbing **R**

      Locally optimize **R'**

      If cost(**R'**)**<**cost(**R**), set **R **= **R'**

      (Otherwise, **R **remains unchanged.)

   End repeat

   Output **R **as the final result

At beginning, a random layout **R **is set as the initial state, then the algorithm optimizes **R **through a neighborhood-test procedure that repeatedly tries to move every single node to its adjacent vacant sites to lower down the cost score. As neighborhood-test proceeds, the layout eventually arrives at such a state that its quality cannot be further improved by moving any single nodes, i.e., the cost function attains a local minimum. To fully optimize **R**, the layout should be managed to escape from the local minimum. For this reason, the algorithm perturbs the layout by moving each node with a given probability *p *to a randomly chosen neighboring location. The perturbed layout is then set to the neighborhood-test procedure. When this re-optimization-after-perturbation process repeats sufficiently many times, the layout becomes hopefully satisfactory and the whole computation ends.

An important feature of the algorithm is that it uses a simple global search strategy relies on the perturbation probability *p*. When *p *= 0, no node is perturbed, the output layout remains unchanged. When *p *= 1, all nodes change their positions, the output layout is little related to the input. For 0<*p *< 1, some parts of the input layout are unchanged, or "memorized". Heavy perturbations (i.e., perturbations with large *p*) lead to significant losses of previous optimization efforts, and consequently the re-optimization will demand relatively high computational expense. In practice, however, the performance is not very sensitive to *p*; moderate values, say, 0.3-0.7, work usually well. In LucidDraw, the default value of *p *is set to 0.7.

Generally, computation speed and layout quality are largely controlled by *niter*, the number of iterations. A small *niter *is obviously preferred for computation speed but usually results in relatively low quality of layouts. Though layout quality benefits from more iterations, very large *niter *is usually not necessary because as the optimization proceeds, better layouts are harder and harder to obtain by re-optimization-after-perturbation. To balance effort and gain, the whole layout process should stop when search efficiency becomes very low. In practice, a moderate value of *niter *= 60 is usually enough to generate satisfied layouts.

### Computational complexity

**Figure 2:**
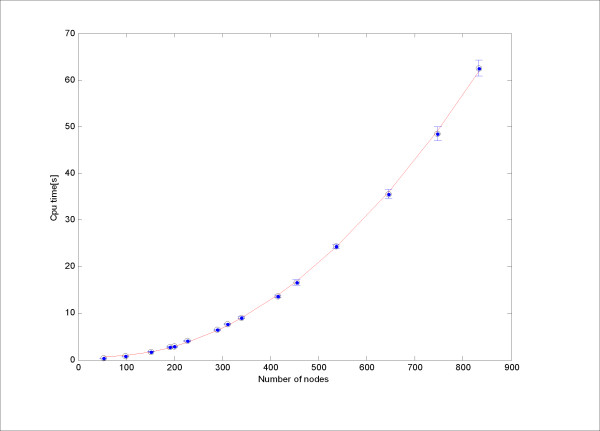
**Computation time of the proposed layout algorithm**. The computation time for each test network was averaged on 30 runs on a common desktop computer with an AMD 5200+ processor. The error bars show the standard deviations. The red line is the fitted quadratic curve.

The accurate complexity of the whole layout process is difficult to estimate analytically. We used a set of example networks to empirically measure the time complexity under the default parameter setting of the algorithm. The results are shown in Figure 2, where the fitted curve is quadratic with respect to the number of nodes, i.e., the required time is *O*(*n*^2^).

### The graphical user interface

**Figure 3:**
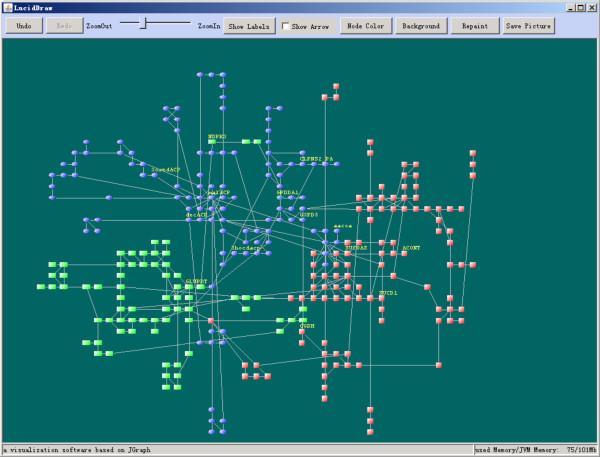
**An overview of LucidDraw GUI**.

**Figure 4:**
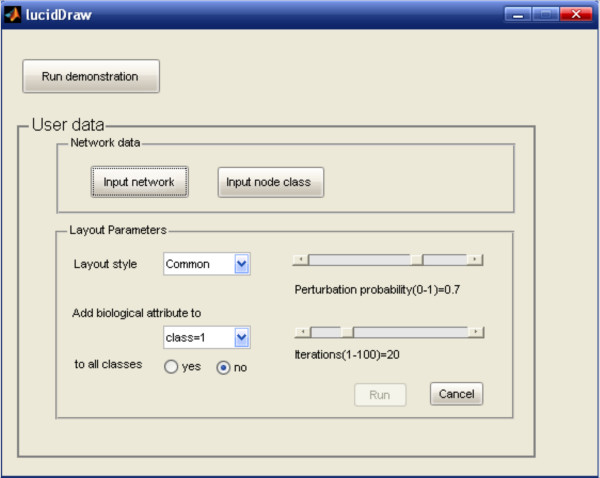
**A demonstration MATLAB GUI making use of LucidDraw**. The user can import network data and change algorithm parameters through this GUI.

The GUI of LucidDraw (Figure 3) is developed based on JGraph http://www.jgraph.com/jgraph.html, an open source graph visualization library written in Java. With the help of abundant graphical functionalities provided by JGraph, LucidDraw supports interactive operations on the network drawings such as moving nodes, zooming in/out, showing/hiding labels or edge arrows. Editing functions like redo/undo are also accessible to make LucidDraw more user-friendly. To aid easy use of LucidDraw in MATLAB environment, we developed another simple GUI (Figure 4) to provide users an intuitive way to manipulate input network data and change detailed parameters of the layout algorithm.

### Treatment of node labels

Node labels are necessary to comprehend network structures shown graphically. To display labels appropriately is not trivial because for drawings of large biochemical networks, room for labels is limited and hence incautious label placement usually causes additional visual complexity. It is usually not satisfied to show all labels simultaneously due to overlaps of labels and nodes. Barsky *et al*. [9] use a greedy method to select as many as possible labels to display without label overlaps, featuring an advantage that more labels are shown at higher zoom levels.

In this work we use three kinds of labels to avoid increasing much visual complexity while making desired node information readable. The first kind is the engraved labels that are shown within the node symbols if the space is large enough. The second kind is the floating labels. A floating label is automatically shown when the mouse pointer is hovering over a node, and disappears when the mouse is moved away. The third kind is the mandatory labels that are statically shown for the right-clicked nodes, staying displayed until the zoom level is changed or the "clear labels" button is pressed.

**Figure 5:**
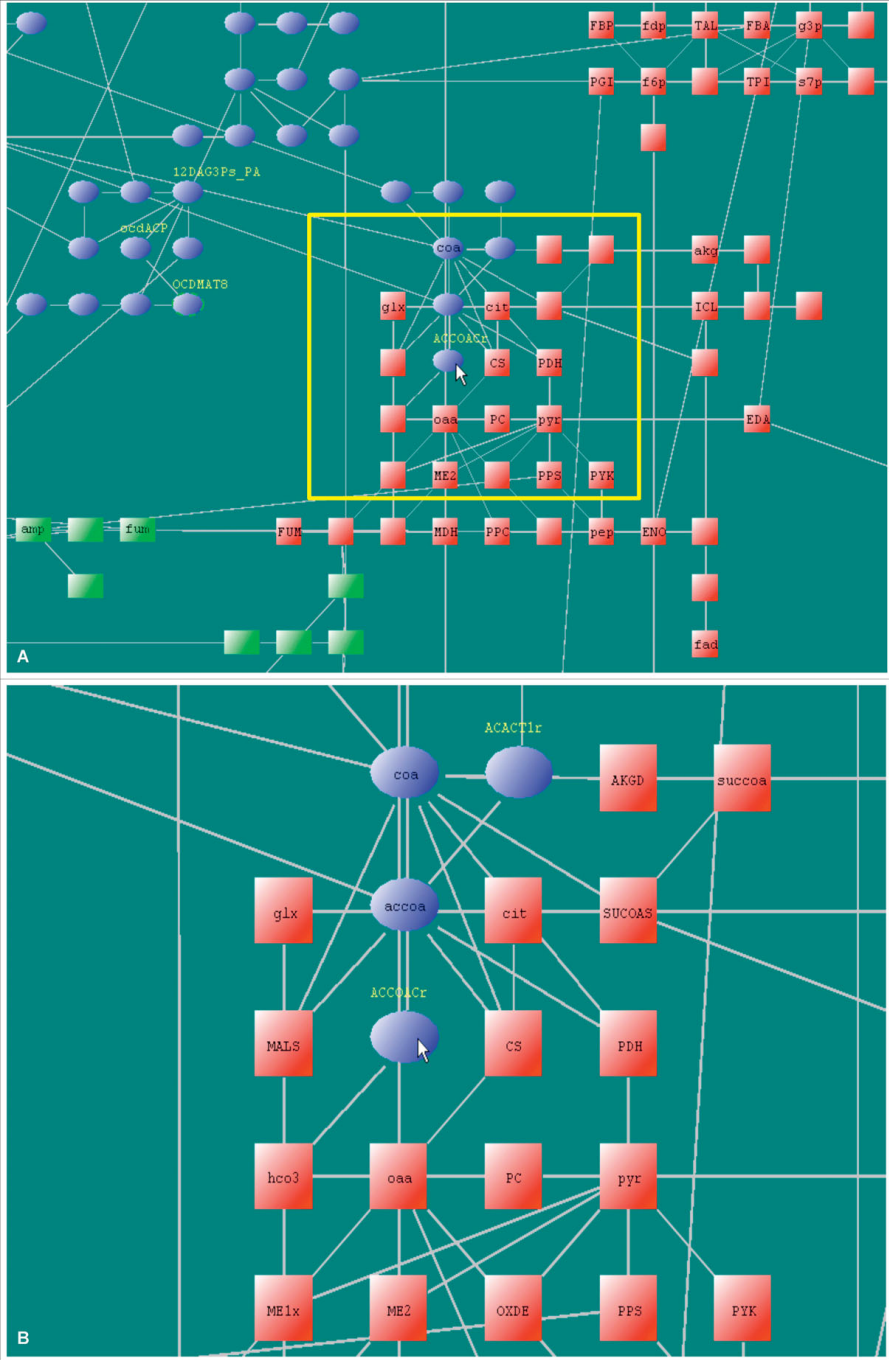
**Three types of labels used in LucidDraw**. LucidDraw uses three kinds of labels to display node names. A) Labels at a relatively low zoom level. Engraved labels are displayed within the symbols of nodes except those with long names. Nodes with label lengths exceeding the symbol size can be shown by mandatory labels outside the nodes, such as "12DAG3Ps_PA" and "ocdACP". The label "ACCOACr" is shown as a floating label when the mouse moves on the node. B) The area marked in A) at a higher zoom level. With the node size increasing, more engraved labels are displayed and fewer mandatory labels are needed.

Displaying of engraved labels is dependent on the zoom level. At higher zoom levels, node symbols become larger and more inside space is available to accommodate longer node names, so there are more node names appearing as engraved labels. Engraved labels can save space but are confined by the node sizes, which cannot label nodes with long names at relatively low zoom levels. Floating labels can make up this deficiency and they do not overlap with other nodes. Mandatory labels are useful when several interesting nodes have long names and cannot be simultaneously displayed at current zoom level by engraved or floating labels. Please see Figure 5 for examples of the three kinds of labels.

## Results

For maximal computation speed, the layout algorithm was implemented in C++ and compiled into a .mexw32 file to work in MATLAB. The GUI for displaying layout results and controlling drawing details were written in Java based on the JGraph library. All executables can be used seamlessly in conjunction with MATLAB with a few auxiliary MATLAB programs, providing users a convenient way to visually analyze complex networks.

### Network data and example layouts

**Figure 6:**
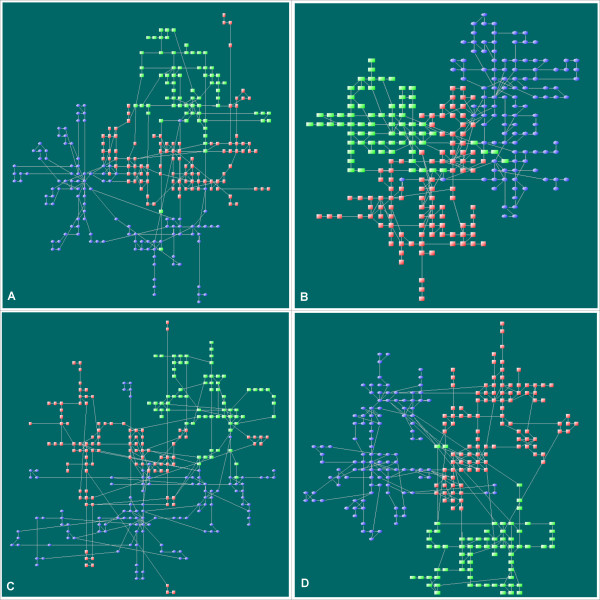
**Example drawings of a typical network**. The network consists of three functional modules of *P. aeruginosa *PAO1 with totally 290 nodes. Nodes of the same modules are drawn in identical shapes and colors: red square = central metabolism; blue ellipse = lipid synthesis; green rectangle = nucleotide synthesis. For clarity, node labels are not shown. Panels A, B, and C correspond to common, compact, and stretched layout styles, respectively. Panel D shows a result when functional module information is supplied in advance. We added an extra positive value (= 10) to the weights *w*_*ij *_of any two nodes which belong to the same modules when graph distances *L*_*ij *_= 2 or 3. The running time for one layout is 6 sec on an AMD 5200+ desktop computer.

The networks used in this work were built from a set of metabolic reactions that are taken from a reconstructed genome-wide metabolic network of *P. aeruginosa *PAO1 [17]. Similar to the method in [18], we converted the set of reactions to a bipartite graph in which metabolites and enzymes are the two classes of nodes. To avoid unnecessary visual complexity caused by a few common molecules, we excluded the currency metabolites such as H_2_O and CO_2_. Due to the space limitation for one-screen figures, we chose 3 modules, central metabolism, lipid synthesis, and nucleotide synthesis from the whole network as examples. LucidDraw outputs for the network with 290 nodes in different layout styles are shown in Figures 6 (A-C). As an illustration to make use of extra biological information, Figure 6(D) is drawn with given modular information where the weights are modified to force nodes of the same metabolic pathways to aggregate together.

### Network analysis with the help of LucidDraw

**Figure 7:**
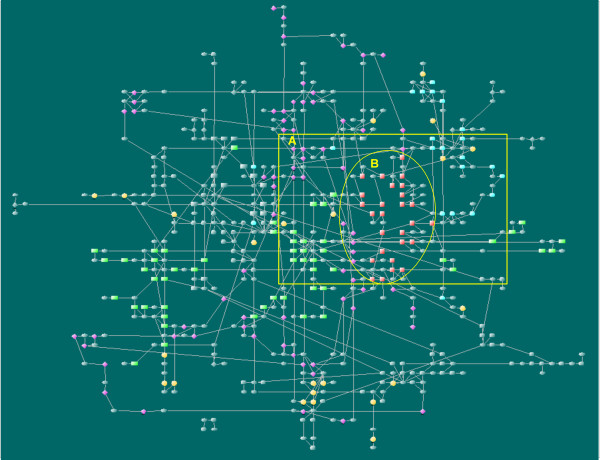
**A LucidDraw layout of a large network with 677 nodes**. The network consists of 7 functional subsystems and some unassigned reactions of *P.aeruginosa *PAO1 with totally 677 nodes. Reaction nodes of the same functional subsystems are drawn in identical shapes and colors: red square = virulence factor synthesis; dark gray round rectangle = central metabolism; light blue round rectangle = sulfur metabolism; green rectangle = lipid synthesis; dark blue rectangle = ethanol/pyruvate metabolism; red diamond = cell wall/LPS synthesis; dark blue diamond = tRNA synthetases; yellow circle = unassigned reactions and dark blue ellipse = metabolites. We added an extra positive value (= 15) to the weights *w*_*ij *_of any two reactions which belong to the virulence factor synthesis. The details of rectangular area A are shown in Figure 8. The area B is mainly occupied by virulence factor synthesis functional subsystem.

We use a 677-node network of *P. aeruginosa *PAO1 model as example to demonstrate the usefulness of LucidDraw. The example network consists of 7 functional subsystems including central metabolism, lipid synthesis, cell wall/LPS synthesis, virulence factor synthesis, tRNA synthetases, ethanol/pyruvate metabolism, sulfur metabolism. Some predicted reactions with subsystem unassigned are also included. The virulence processes of *P. aeruginosa *are of great importance from the view point of medical applications. In the map (Figure 4(a) in [17]) drawn manually by the authors who constructed the network, the reactions of the virulence subsystem scattered in separated regions; besides, some related metabolites are represented by two or more graphical symbols, i.e., a metabolite may have several different positions. This brings difficulty to grasp overall characteristics of the relations between virulence processes and other subsystems. Obviously, producing a new map by hand with desired properties needs much effort and is practically unfeasible. In such a case, by means of LucidDraw, it is easy to generate a drawing to highlight the relations between the focused subsystems. To do so, we add an additional weight to the each pair of reaction nodes if they both belong to the same subsystem. In the resulted layout, the virulence associated nodes are positioned in adjacent locations, as shown in Figure 7. The figure intuitively displays that the virulence processes are closely related to "cell wall/LPS synthesis", as well as "lipid synthesis" and "sulfur metabolism" subsystems. The observations gained through LucidDraw are consistent with previous researches [19-21]. The reason is that the major metabolic precursors in virulence processes such as UDP-N-acetyl-D-glucosamine (uacgam), dTDP-4-dehydro-6-deoxy-L-mannose (dtdpddm), and Acetyl-ACP (acACP), are also involved in cell wall/LPS synthesis and lipid synthesis.

**Figure 8:**
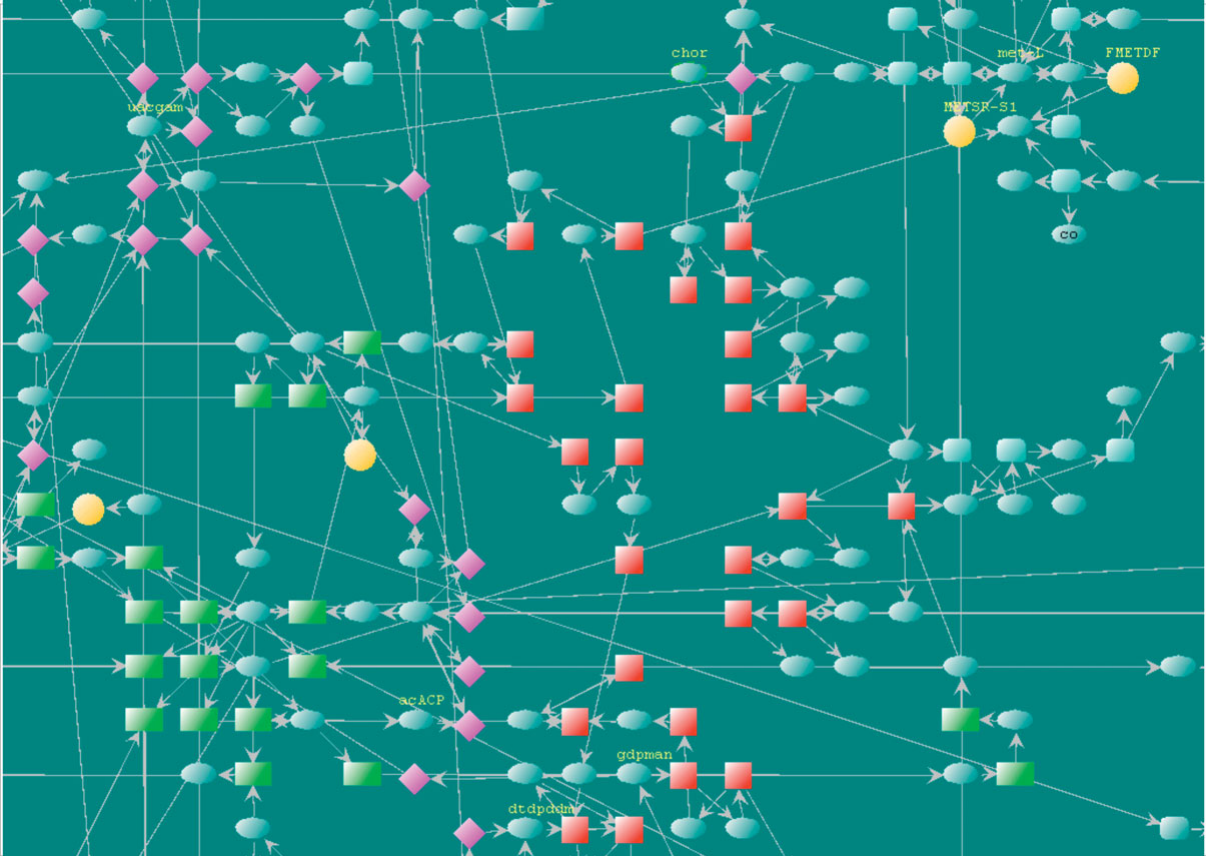
**A close-up of the layout region containing virulence factor synthesis and related functional subsystems**. The major metabolic precursors in virulence processes UDP-N-acetyl-D-glucosamine (uacgam), Acetyl-ACP (acACP), dTDP-4-dehydro-6-deoxy-L-mannose (dtdpddm), GDP-D-mannose (gdpman), and Chorismate (chor) (marked with mandatory labels) are also important metabolites of closely related functional subsystems. The subsystem-unassigned reactions FMETDF and METSR-S1 are shown to have close connectivity with sulfur metabolism (top right corner).

From Figure 8 (a close-up of the marked area in Figure 7) we can also see close relationships between some unassigned reactions and certain functional subsystems. For example, the reactions FMETDF (formylmethionine deformylase) and METSR-S1 (L-methionine-S-oxide reductase) sit closely to sulfur metabolism subsystem in the layout. The two reactions are tied to sulphur metabolism through L-Methionine (met-L) which is involved in many processes in the subsystem. This is not apparent without a properly drawn graphical presentation. The intuitive result may provide clues for further investigations to clear the uncertainty in current knowledge.

## Discussion

A good layout algorithm depends on two factors: a proper cost function and an efficient optimization method. LucidDraw adopts a similar cost function as the previous work [7] but a new optimization procedure with much higher efficiency. With the search area of every node reducing drastically, the neighborhood-test method greatly lowers the computational cost. To fully optimize the cost function, the re-optimization-after-perturbation strategy is used to force the layout to escape from current local minimum and search for better layouts. The perturbation strategy, despite its simplicity, achieves rather good performance comparing to other sophisticated heuristics like simulated annealing. The technique was also employed in other discrete global optimization problems [22,23]. Together with the neighbourhood-test approach, the technique speeds up the layout process dramatically and makes it possible for LucidDraw to serve as an instant visualization tool in the context of a wide range of network analysis tasks. The effect of the optimization strategy is substantial. For a network with 677 nodes, our new algorithm takes ~30 sec to generate an acceptable layout; while our previous algorithm [7] needs >3 hr CPU time and a large amount (~1 GB) of memory. Another available grid layout software, Cerebral [3], can produce a layered layout in ~3 min with the prerequisite that all nodes of the entire network are already divided into appropriate groups, and the order of the layers is provided in advance by the user.

For ease of use in case of large networks, LucidDraw provides a comprehensive solution to aid users to get node information conveniently through three kinds of labels. As comparison, other network modeling tools have fewer choices to display node labels. For instance, Cytoscape [10], VisAnt [12], and YANAsquare [24] use two labeling methods (engraved and floating); VANTED [25] uses only engraved labels.

In LucidDraw, we design more flexible weight matrices and provide three elaborated evaluation schemes of the weight matrix through extensive experiments. Compared to previous work implemented for network modeling software, LucidDraw also provides flexibility to make customized drawings to aid visual network analysis with the help of the powerful numeric capabilities of MATLAB.

LucidDraw does not depend on predefined module information to produce layouts with nodes belonging to the same modules located closely (Figures 6(A-C)). This does not exclude the possibility to use the module data; instead, such data are easy to incorporate through modifying the weights to force nodes to distribute with desired position propensities (Figure 6(D)).

It should be noted that some network modeling software such as Cytoscape [10] and VANTED [25] provide grid based visualizations, but the underlying layout methods are obviously different from ours. For comparison, please refer to Additional file 1. A remained issue of LucidDraw is the edge-node crossings which occur occasionally but indeed confuse the relations between a few nodes. To relieve the problem, Miyano and co-workers introduced penalty terms in the cost function [4,5] at the expense of higher computational complexity. Another feasible choice is to use curved edges [3]. It should be noticed that a thorough solution of the edge-node crossing problem must take node sizes into account, which is a future direction of this work.

## Conclusions

We present a MATLAB tool, LucidDraw, to meet the needs of convenient visulization of complex biochemical networks. The tool is fully accessible within MATLAB and capable of drawing typical networks in seconds with appropriately separated modules in a compact space. Users can control layout styles, drawing details, as well as extra biological attributes to get sufficiently customized drawings.

## Availability and Requirements

- **Project name: **LucidDraw

- **Project home page**: http://bioinf.jiangnan.edu.cn

- **Operating system (s)**: Windows (32bit version)

- **Programming language**: Java, C++

- **Other requirements**: MATLAB 7.5 (32bit version), Java 1.6

- **License**: Free for non-commercial use.

The LucidDraw programs and sample data are given in Additional file 2. A demonstration video is provided in Additional file 3. Latest software and more example networks can be found at http://bioinf.jiangnan.edu.cn.

## Authors' contributions

SH and WL designed the project and developed the programs. JM, GS, and ZW took part in design, data collection, and software evaluation. SH and WL wrote the paper. All authors read and approved the final manuscript.

## Supplementary Material

Additional file 1 **Example network drawings by LucidDraw and other software**. Figures in Additional file 1 are drawings of the same network, YeastGlycolysisJDClean which was taken from VANTED http://vanted.ipk-gatersleben.de/. Click here for file

Additional file 2 **The LucidDraw software**. The LucidDraw.zip package includes the following files: glayoutdll.mexw32 (the layout computation program); LucidDraw.jar (the Java package for LucidDraw GUI); Jgraph.jar (the JGraph library); lucidDraw.m, lucidDraw.fig, FastGridLayout.m, glweight.m, Read_Classid.m, LayoutView.m, Text_To_AdjacencyMatrix.m (the MATLAB scripts for using LucidDraw); PAO1_290nodes.txt, PAO1_290nodes_class.txt (an example network). In MATLAB, run lucidDraw.m. Click here for file

Additional file 3 **The LucidDraw demo video**. Double-click LucidDrawDemoVideo.exe to watch the video. Click here for file
