# Supplementary material for: LucidDraw: Efficiently visualizing complex biochemical networks within MATLAB
Source: BMC Bioinformatics. 2010 Jan 15;11:31. doi: 10.1186/1471-2105-11-31 (PMC2825225; doi:10.1186/1471-2105-11-31)
Supplement: Additional file 1 — Example network drawings by LucidDraw and other software. Figures in Additional file 1 are drawings of the same network, YeastGlycolysisJDClean which was taken from VANTED http://vanted.ipk-gatersleben.de/. [file 1471-2105-11-31-S1.PDF]

Example network drawings by LucidDraw and other software

Figures 1-6 are drawings of the same network, YeastGlycolysisJDClean which was taken from VANTED (http://vanted.ipk-gatersleben.de/).

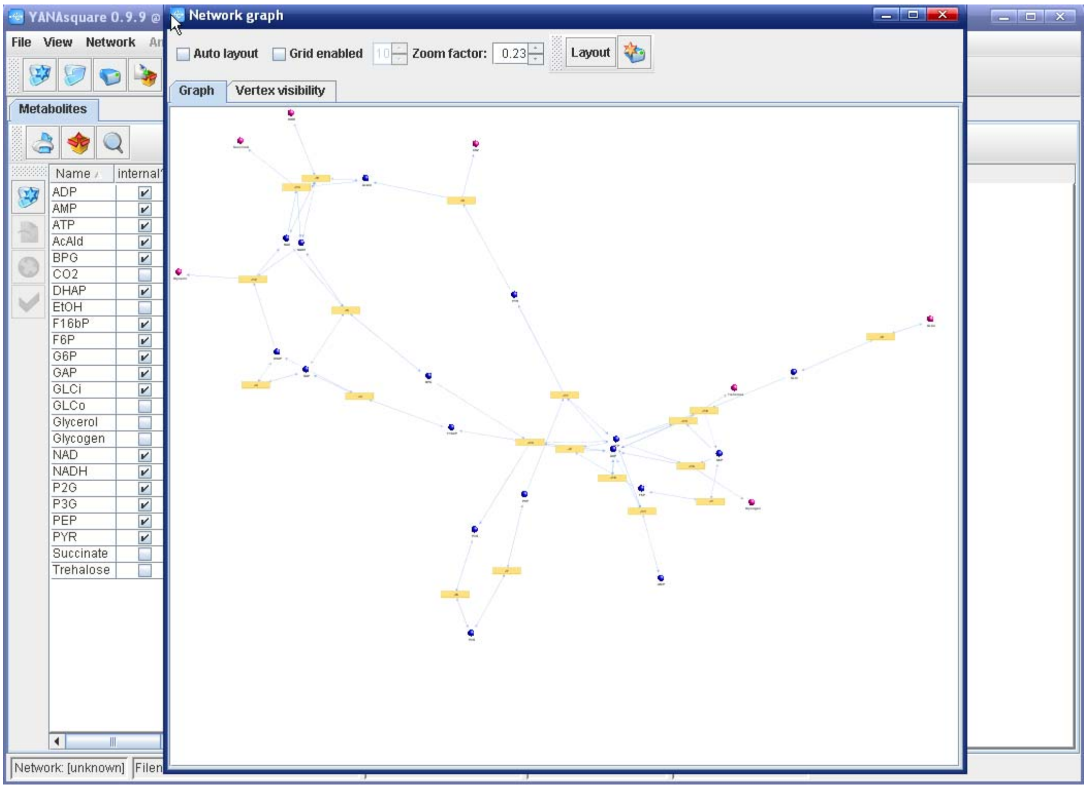

Figure 1. YANAsquare, spring-embedding.

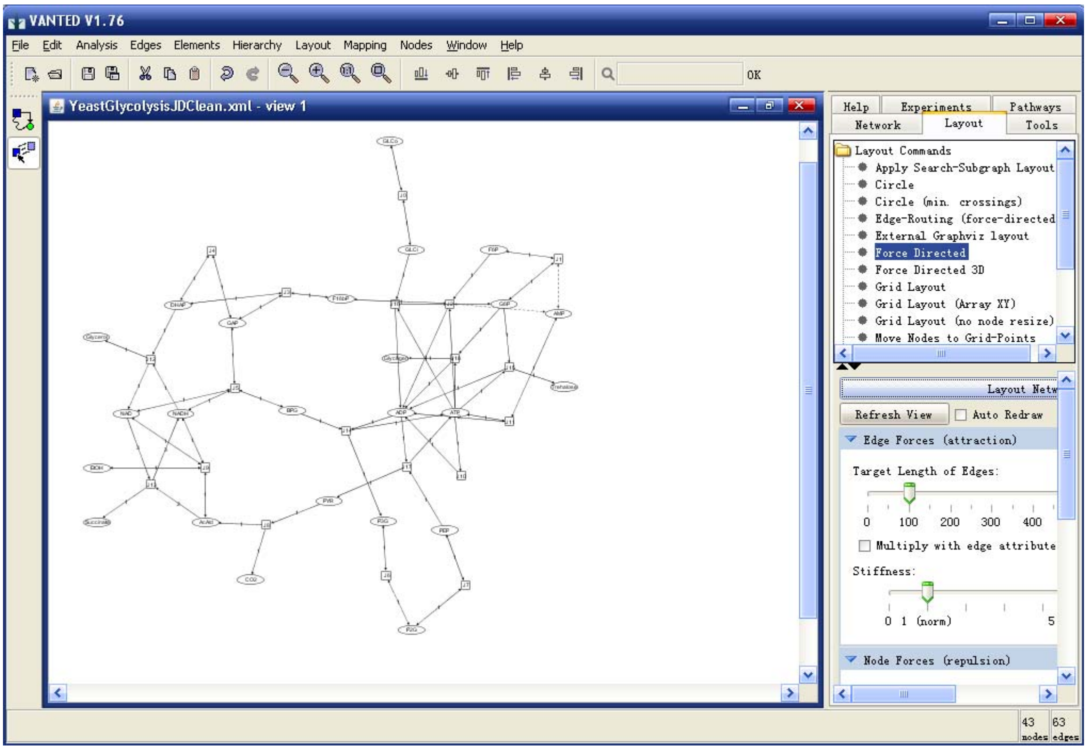

Figure 2. VANTED, force-directed.

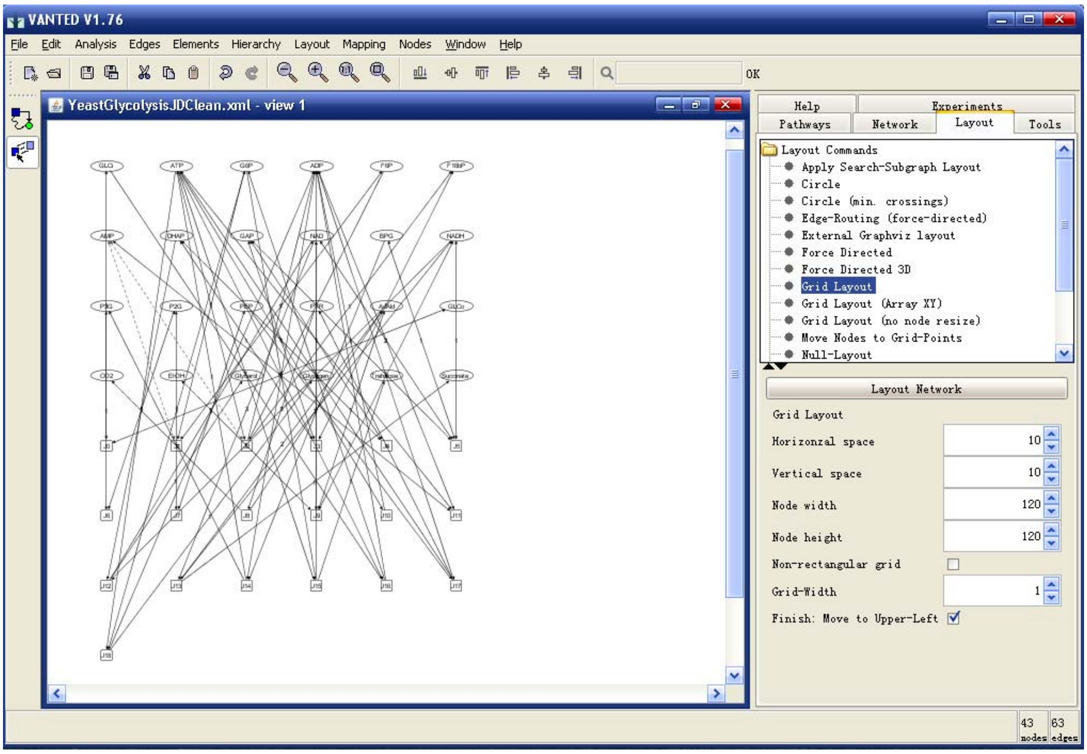

Figure 3. VANTED, grid layout.

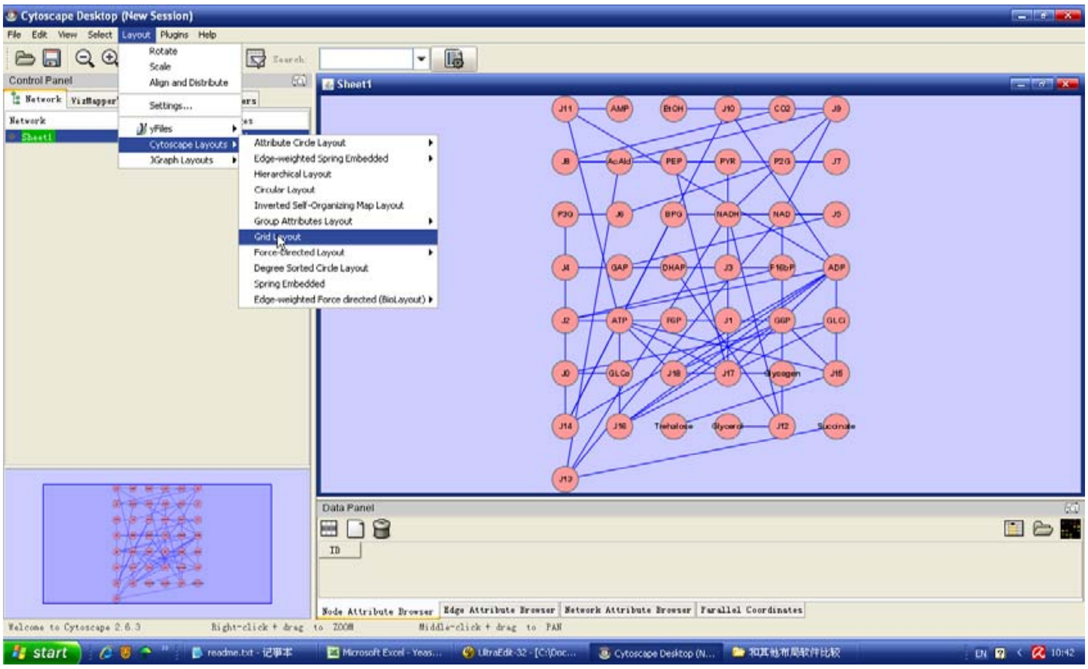

Figure 4. Cytoscape, grid layout.

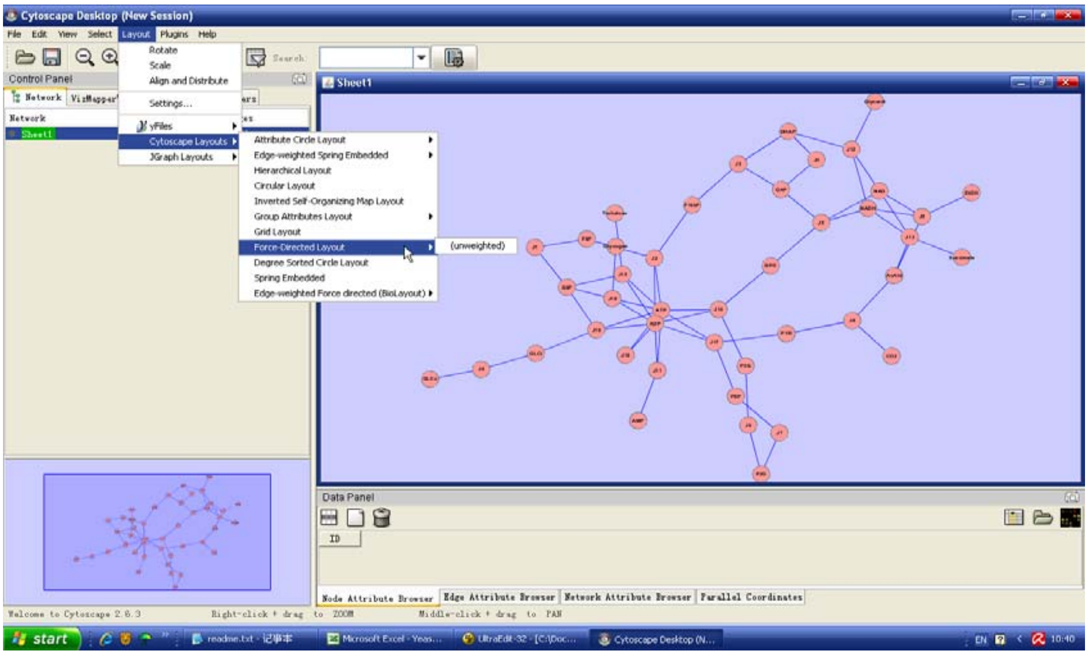

Figure 5. Cytoscape, force-directed (unweighted).

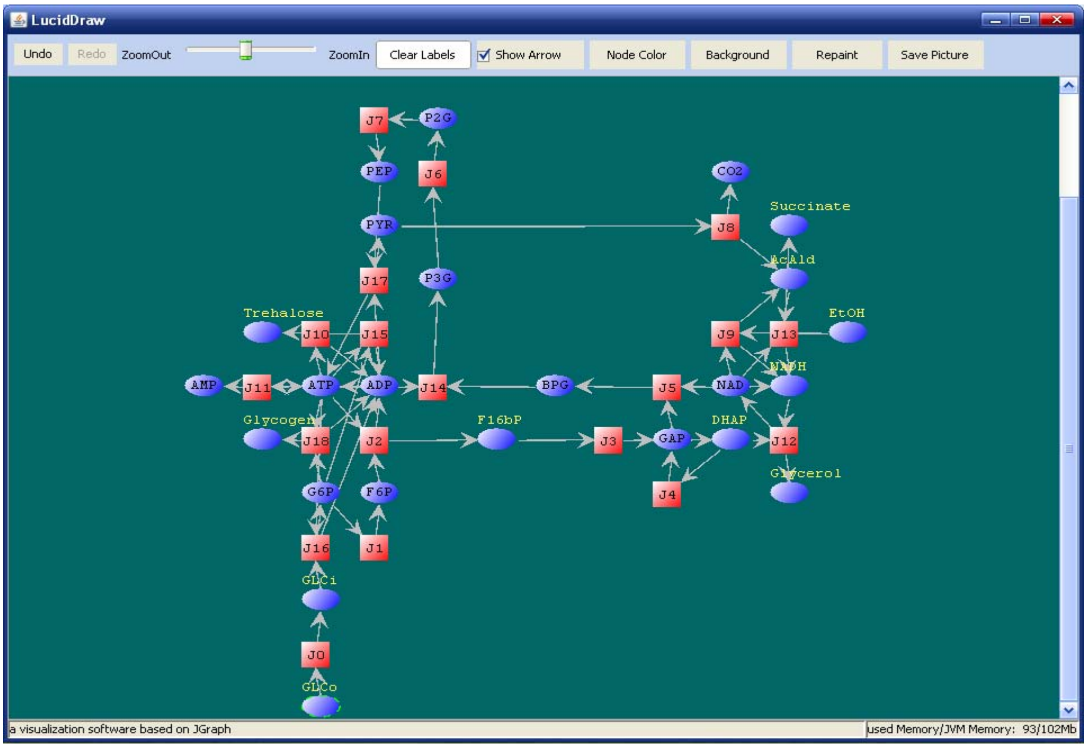

Figure 6. LucidDraw, grid layout.
